# Investigating the Efficacy and Experiences With Narrative Exposure Therapy in Severe Mentally Ill Patients With Comorbid Post-traumatic Stress Disorder Receiving Flexible Assertive Community Treatment: A Mixed Methods Study

**DOI:** 10.3389/fpsyt.2022.804491

**Published:** 2022-04-28

**Authors:** Maria Mauritz, Peter Goossens, Ruud Jongedijk, Hester Vermeulen, Betsie van Gaal

**Affiliations:** ^1^GGNet Center for Mental Health Care, Warnsveld, Netherlands; ^2^Radboud University Medical Center, Radboud Institute for Health Sciences, IQ Healthcare, Nijmegen, Netherlands; ^3^Dimence Group, Center for Mental Health Care, SCBS Bipolar Disorders, Deventer, Netherlands; ^4^Department of Public Health, Faculty of Medicine and Health Sciences, University Center for Nursing and Midwifery, Ghent University, Ghent, Belgium; ^5^ARQ Centrum’45, Oegstgeest, Netherlands; ^6^ARQ National Psychotrauma Centre, Diemen, Netherlands; ^7^School of Health Studies, HAN University of Applied Sciences, Nijmegen, Netherlands

**Keywords:** PTSD, severe mental disorder, flexible assertive community treatment (FACT), therapist, nurse, qualitative study, mixed methods, narrative exposure therapy (NET)

## Abstract

**Background:**

Patients with severe mental illness with repeated interpersonal trauma and post-traumatic stress disorder (PTSD) have a negative illness progression. Traumas are often not treated because of their vulnerability. Narrative exposure therapy (NET) is an effective trauma therapy. It is unknown whether NET is effective and tolerable in these patients receiving community mental healthcare.

**Objectives:**

The objectives of this study are (1) to gain insights into patients’ experiences before, during, and after NET concerning changes in PTSD, dissociative and severe mental ill symptoms, care needs (CAN), quality of life, and global functioning; (2) to identify factors that influence diagnostic changes after NET as compared to patients’ experiences. These insights will help to decide whether NET should be incorporated in usual care for these patients.

**Design:**

A mixed methods convergent design consists of a grounded theory approach with thematic analysis followed by a merged analysis, comparing quantitative, and qualitative data for each participant and by means of a joint matrix.

**Participants:**

Adult psychiatric outpatients (age, 21–65) with post-traumatic stress disorder (PTSD) related to repeated interpersonal trauma were indicted for the study.

**Methods:**

Baseline demographics and clinical characteristics were assessed. Qualitative data were collected 3 months after NET using individual semi-structured in-depth interviews. The merged analysis compared quantitative and qualitative results for each participant.

**Results:**

Twenty-three outpatients (female, 82%) with a mean age of 49.9 years (SD 9.8) participated in the study. Participants experienced NET as intensive, and most of them tolerated it well. Afterward, eighteen participants perceived less symptoms. Mixed analysis showed substantial congruency between quantitative scores and participants’ perceptions of PTSD, dissociative symptoms, and CAN (Cohen’s kappa > 0.4). Remission of PTSD was associated with sufficient experienced support.

**Conclusion:**

Outpatients with severe mental illness underwent intensive NET, and most of them tolerate it well. This therapy is clearly efficacious in this group.

**Clinical Trial Registration:**

[www.ClinicalTrials.gov], identifier [NL5608 (NTR5714)].

## Introduction

The attention given for the high prevalence rates of physical abuse (47–65%), sexual abuse (36–37%), and post-traumatic stress disorder (PTSD) (30–34%) in patients with severe mental illness (SMI) is still growing (1–3). The need for adequate diagnostics and treatment is increasingly recognized because trauma and PTSD have a negative influence on the course of SMI, including an increased risk of poor physical health; addiction; problems with social, occupational, and community functioning; reduced quality of life; and criminalization (1, 4–6).

Post-traumatic stress disorder has been also associated with substantial medical and economic burden. Recent public health preventive innovations have included integrated medical and behavioral healthcare, such as modifications of trauma-focused treatment (TFT), the use of novel and augmentative psychopharmacological agents, and the use of technology. Research on the impact of traumatic stress, as well as prevention strategies for PTSD, has resulted in an improved understanding of its impact and more effective public health interventions (7).

Trauma-focused cognitive behavior therapy is the best validated treatment for PTSD, but it has stagnated over recent decades, and only two-thirds of patients with PTSD adequately respond to this intervention. Globally, most people with PTSD do not access evidence-based treatment, and this situation is much worse in low- and middle-income countries. Bryant advocates better management so that more patients can benefit from TFT (8).

Studies have shown that TFT, such as prolonged exposure, cognitive behavioral treatment, and, eye movement desensitization reprocessing (EMDR), have proved to be effective for patients with major depressive, bipolar, and psychotic disorders with comorbid PTSD (3, 9–12).

Narrative exposure therapy is a relatively new TFT (13). NET is suited for people who are exposed to repeated traumatic events during the course of their life. NET has a lifetime perspective, integrating an exposure-based narration of traumatic experiences together with an elaboration of these experiences in the autobiographical context.

NET appeared effective in vulnerable, traumatized groups, like refugees, child soldiers, patients with early childhood trauma, and other vulnerable, traumatized groups, among which are patients with tendency for dissociation, major depressive disorder, or borderline personality disorder, and is also suitable for children and older adults (14–16). Until now, NET has not been specifically investigated in outpatients with SMI, and, to our knowledge, there are no qualitative and mixed studies on NET (15, 17).

In a mixed methods study consisting of a quantitative and qualitative strand, NET was offered to outpatients with SMI with comorbid post-traumatic stress disorder associated with repeated interpersonal trauma, and they received flexible assertive community treatment. This Dutch variant of community mental healthcare consists of coordinated multidisciplinary treatment interventions for outpatients with SMI, including collaborative care, pharmacotherapy, case management, crisis interventions, and outreaching nursing care (18).

In another article, we described quantitative results, showing that diagnostic PTSD scores decreased from a mean of 37.9 before NET (T0) to 31.9 after post-treatment (T1) (–6.0, 95% CI –10.0 to –2.0) and to 24.5 at follow-up (T2) (–13.4, 95% CI –17.4 to –9.4). Dissociation, psychiatric symptoms, contacts, and medication decreased, global functioning increased, and quality of life and perceived needs did not significantly change (19).

The purpose of the present study is to evaluate outpatients with NET in SMI by a qualitative analysis of patient perceptions and to compare this to the quantitative assessment reported before.

We are working on another study that analyzes the interviews by full grounded theory on the meaning of NET.

## Objectives

The objectives of this study are (1) to gain insights into patients’ experiences before, during, and after NET concerning changes in post-traumatic stress disorder, dissociative and severe mental ill symptoms, care needs (CAN), quality of life, and global functioning and (2) to identify factors that influence the results of NET in terms of diagnostic changes compared with patients’ experiences. These insights will help in balanced decision-making on whether NET should be incorporated in usual care for these patients (20).

## Materials and Methods

### Design

The intervention was evaluated by means of a convergent mixed methods design, including a quantitative and a qualitative strand (21–23). The quantitative strand had a single-group pre-test-post-test repeated-measures design and was published elsewhere (19). The qualitative strand was based on a grounded theory approach and consisted of individual semi-structured in-depth interviews with all participating patients. The present integration of these strands focuses on interpreting how qualitative results enhance the understanding of quantitative outcomes in a one-to-one analysis.

This study was registered in The Netherlands National Trial Register, Trial NL5608 (NTR5714) registration date: 2016-02-18; first recruitment: 2016-03-15. The Committee on Research Involving Human Subjects, Arnhem-Nijmegen, provided ethical approval (No. 1843–2015). The study was carried out in a mental health center in the Netherlands from April 2016 to January 2019.

### Participants: Outpatients With Severe Mental Illness With Comorbid Post-traumatic Stress Disorder

The target population consisted of adult outpatients with SMI (age, 21–65) who received flexible assertive community treatment in a community mental healthcare context. Due to the high prevalence of trauma exposure and post-traumatic stress disorder, outpatients with SMI were screened for traumatic events and the presence of post-traumatic stress disorder. Patients with comorbid post-traumatic stress disorder and a history of repeated interpersonal trauma were eligible for NET.

The inclusion criteria were outpatients with (1) SMI, defined as the presence of a bipolar, major depressive, schizophrenia-spectrum disorders according to the Mini-International Neuropsychiatric Interview (M.I.N.I.-plus) or the presence of a personality disorder according to the Structured Clinical Interview for DSM-IV Personality Disorders (SCID-II), with reduced global functioning according to the Global Assessment of Functioning (GAF)-score < 60 during 2 or more years according to chart diagnosis; (2) a trauma history including repeated physical and/or sexual abuse according to the Life of Events Checklist for DSM-5 (LEC-5); and (3) the existence of post-traumatic stress disorder according to the Clinical-Administered Post-traumatic Stress Disorder Scale for DSM-5 (CAPS-5).

The exclusion criteria were outpatients with (1) the provision of other TFT within 12 months prior to the study; (2) antisocial personality disorder; (3) dissociative identity disorder; and (4) the provision of involuntary treatment following the Dutch Mental Health Law.

### Inclusion

NET was first indicated by multidisciplinary consultation in the flexible assertive community treatment team. Patients received oral and written information about NET from the therapist. When the patients decided to accept NET, the therapist asked them to participate in the study. Written information about the entire study was provided by the researcher (the first author). After 1 week, the patients were called by the researcher to verify whether the information was well understood. If so, patients were asked to provide oral and written consent. The participants received the following information about the researcher: a female nurse practitioner and a scientist working at the mental health center but who were not involved in patients’ flexible assertive community treatment. The researcher had no relationship with the participants prior to the commencement of the study, except for one female patient who had received flexible assertive community treatment from the researcher 4 years earlier.

### Intervention

NET was conducted according to the Dutch manual (24, 25), which is based on Schauer et al. (26), and consisted of weekly 90-min sessions with a minimum of five and a maximum of sixteen sessions. During NET, flexible assertive community treatment was continued with minimal biweekly supportive interventions by the community treatment team members, mainly nurses. This was asked as an important condition because of the vulnerability of outpatients with SMI.

NET was provided by five therapists, consisting of three nurse practitioners and two clinical psychologists who were recruited from different flexible assertive community treatment teams at the Mental Health Center and were certified as NET-therapists after following a 3-day NET training by qualified trainers in 2015–2016. They completed additional group video supervision in ten 90-min sessions by a trained NET-supervisor during the study (27).

### Research Team and Reflexivity

The age of the team members was given at the start of the analysis in January 2019. The first author, female and aged 53, is a nurse scientist (MSc) and a nurse practitioner (APRN) in mental healthcare. She performs psychiatric and nursing diagnostics and care, pharmacotherapy, psychological treatment, and mainly works with outpatients with traumatized severe mental condition. She is a certified NET-therapist and experienced in other TFT such as eye movement desensitization reprocessing and prolonged exposure (28). Her academic training included qualitative research skills, and she graduated on a grounded theory study of loss and grief in schizophrenia (29).

The second author, male and aged 54, is a nurse scientist (Ph.D.) and nurse practitioner (APRN) in mental healthcare. He is a specialist in bipolar disorders and other severe mental disorders. He is also a part-time professor in Health Sciences and experienced in qualitative, quantitative, and mixed models approaches.

The third author, male and aged 59, is a psychiatrist (MD). He introduced NET in the Netherlands and is a certified NET therapist, teacher, and supervisor (24).

The fourth author, female and aged 50, is a clinical epidemiologist (Ph.D.), a full professor in Nursing Science and has extensive experience in quantitated and qualitative research. She supervised the whole study.

The fifth author, female and aged 54, is a nurse scientist (Ph.D.) and registered nurse (RN) in an academic hospital care. She is also a senior lecturer and senior researcher in applied nursing sciences and has experience in quantitative, qualitative, and mixed methods approaches.

### Data Collection

In this convergent design, quantitative and qualitative data were concurrently collected before merging the data (22).

#### Demographics and Clinical Characteristics

Demographic data were collected in a former study *via* the electronic patient record at the baseline and included gender, age, marital status, cultural background, education, living condition, and employment. Clinical characteristics were collected with the M.I.N.I-plus and comprised the primary SMI diagnosis, duration of illness, number of suicide attempts, suicide risks, and substance abuse (19).

#### Quantitative Measurements

Quantitative measurements used in this study were assessed as described before (19, 20). In short, six quantitative measures of outcomes were used: post-traumatic stress disorder (CAPS) (30), dissociative symptoms (DES) (31), severe mental ill symptoms (HoNOS) (32), CAN (33), quality of life (MANSA) (34), and global functioning (GAF) (35). These outcomes measured were pre-treatment (T0), 1 month (T1) after treatment, and 7 months (T2) after treatment. Care consumption (prescribed medication, number of therapeutic contacts, and duration in minutes) was collected *via* the electronic patient record from 6 months prior to T0 until 7 months after T2 (19).

#### Semistructured in-Depth Interviews

Semistructured in-depth interviews were held 3 months post-treatment (T1) and lasted at most 60 min. The participants were allowed to choose to have the interview at their home or in the local Mental Health Unit center. Supervised by the second author (PG), the first author (MM) developed a topic list and an interview guide for the semistructured interviews. Based on the pre-arranged sensitive concepts in the study protocol, perceived changes in post-traumatic stress disorder, dissociative and severe mental Ill symptoms, CAN, and quality of life over time, a topic list, and an interview guide were developed for the semistructured interviews by MM and supervised by PG. The interview guide was adjusted two times when new relevant topics emerged in the interviews; Box 1 provides the final topic list and Supplementary Material 2 provides the final interview guide. MM conducted the interviews and was blinded to the quantitative measurement results to avoid bias based on foreknowledge. Each interview was held with a *neutral attitude.* All interviews were audiotaped and transcribed verbatim. PG provided written feedback on each transcript after listening to the recordings. Then, MM and PG reflected on the interview style, and they explored new themes and evaluated the interview guide. To provide *member checks*, the transcripts were summarized by MM and sent to the participants. After 1 week, MM contacted the participants by telephone to verify whether the summary was complete and accurate.

**Box 1. Topic list.**

Topics derived from and arranged by each sensitizing concept

*Experienced severity of symptoms **before, during and after** NET*

• PTSD
• Symptoms of psychotic, bipolar, or depressive disorder
• Suicidality

*Changes in care needs*

• perceived decreased care needs
• perceived persisting or increased care needs

*Changes in quality of life and daily life functioning*

• perceived quality of life
• perceived effects on daily life functioning

*Influencing factors*

• success
• failure

Mauritz, et al. (19), p. 5.

## Analysis

Qualitative data were analyzed independent of the earlier quantitative analysis (objective 1). Subsequently, the mixed methods analysis combined the quantitative measurements with the qualitative results (Objective 2) to assess (in) congruency between quantitative measurements and qualitative interview data.

### Qualitative Analysis

At the start, the analysis was based on the grounded theory approach to gain more insights into the experiences of the participants. To facilitate the analysis of interview data, the Qualitative Analysis Guide of Leuven was used (36). This guide is specifically used for interview data and distinguishes two parts with each five stages: First, the preparation of the coding process with paper and pencil work and, second, the actual coding process using qualitative software in ten stages of analysis (see Box 2). MM and PG performed all the ten stages, which were carefully documented for *transparency and reproducibility*. After rereading the interviews (1), narrative reports were independently made, compared, discussed, corrected, and integrated (2). A conceptual interview scheme was then derived (3), and a fitting test of the conceptual interview scheme was performed for all interviews by HV, BvG, and RJ. To critically evaluate supporting and contradictory information, *triangulation* included feedback by in-depth discussion with all the authors. Then, corrections and additions were made (4). After *constant comparison* (5), a preliminary list of concepts was drafted (6). Subsequently, Atlas-ti 8 was used to link all relevant fragments to appropriate codes (7). Then, a *thematic analysis* was undertaken with a deductive approach for the following concepts: experienced post-traumatic stress disorder, dissociative and severe mental Ill symptoms, CAN, quality of life, and global functioning. This analysis was aimed at meaning, dimensions, and characteristics (8). Extraction of the essential structure was displayed using an excel file (9) (see Supplementary Material 1), and the essential results were described (10).

**Box 2. The 10 stages of the Qualitative Analysis Guide of Leuven QUAGOL.**

1. Thorough (re)reading of the interviews to receive a holistic understanding of the respondent’s experience
2. Narrative interview report: a brief abstract of the key storylines of the interview
3. From narrative interview report to conceptual interview scheme
4. Fitting-test of the conceptual interview scheme: testing the appropriateness of schematic card in dialogue
5. Constant comparison process: forward-backwards movement between within-case and across-case analysis
6. Drawing up u list of concepts: a common list of concepts as preliminary codes
7. Coding process – back to the ‘ground’: linking all relevant fragments to the appropriate codes
8. Analysis of concepts: description of concepts, their meaning, dimensions & characteristics
9. Extraction of the essential structure: conceptual framework or story-line
10. Description of the results and description of the essential findings

Adapted from Dierckx de Casterlé et al. (36).

### Merged Data Analysis of Quantitative and Qualitative Data

The merged analysis compared the results from the quantitative and qualitative data for each participant by means of a joint matrix, a display that places quantitative results side by side with qualitative themes using Excel 2013 (see Supplementary Material 1).

The results from the two databases were compared for the following specified dimensions**:** Post-traumatic stress disorder, dissociative symptoms, SMI symptoms, CAN, quality of life, and global functioning. The Excel file listed quantitative results in columns for T0, T1, and T2. For each quantitative outcome, a second column was added to represent qualitative results, while quantitative results were hidden.

Next, both quantitative and qualitative results were compared, and a third column was added to indicate whether quantitative and qualitative results were congruent or incongruent.

Subsequently, it was examined how often congruence occurred in participants. This procedure also facilitated *triangulation of different methods* possible. The comparisons were presented in Supplementary Material 1.

Congruence of structured assessment instruments (CAPS-5, DES, HoNOS, CAN, MANSA, GAF) and patients’ perceptions (qualitative interviews) was quantified as the percent agreement and as Cohen’s kappa (37), followed by statistical testing using the Fisher exact test (38).

### Additional Merged Analysis

During the analysis of the narrative reports (second stage, QUAGOL), MM and PG noticed that professional and informal support was, possibly, an important factor for NET results. At the third stage, the concepts “supportive caregivers” and “significant others” were inserted into the final conceptual scheme as influencing factors. In Step 7 (Box 2), we (PG, MM) identified that the patients often mentioned the relevance of support. Subsequently, we decided along with all the authors to investigate a possible relation between perceived care support and whether or not the patients were in remission. The percentage of the patients who were in remission was compared between those who did and those who did not perceive sufficient support, followed by statistical testing (Fisher exact test) (38).

## Results

### Participants: Outpatients With Severe Mental Illness

As described in Table 1, the study population included 23 adult outpatients with SMI (mean age, 49.9 years; SD, 9.8) with comorbid post-traumatic stress disorder associated with repeated interpersonal trauma, i.e., physical and/or sexual abuse in childhood and/or adulthood. The majority of the 23 participants had a Dutch background (*n* = 19) and were female (*n* = 19). The mean duration of post-traumatic stress disorder was 24.1 years (SD 14.5) (19). All the participants received NET in a flexible assertive community treatment context (*n* = 23) of whom 21 completed NET. Two female participants (age, 50 and 63) terminated NET prematurely because they could not tolerate the exposure to traumatic memories. One of them still agreed to participate in the interview. Therefore, a total of 22 interviews were conducted. Results of the quantitative analysis are provided in Table 2.

**TABLE 1:** Demographic and clinical characteristics (*n* = 23).^a^

Demographics			
Age, y	Median, mean, (SD)	49.9	(9.81)
		No	%
Gender	Female	19	82.6
Cultural background	Dutch	18	78.3
	Western non-Dutch	1	4.3
	Non-western	4	17.4
Education	Low	7	30.4
	Middle	14	60.9
	High	2	8.7
Employment	Employed	1	4.3
	Sheltered employed	8	34.8
	Unemployed	14	60.9
Living condition	Married/cohabiting	12	52.2
	Alone	7	30.4
	Sheltered housing	4	17.4
**Clinical characteristics**			
		No	%
SMI diagnosis (MINI-plus)^b^	Schizophrenia spectrum disorder	4	17.4
	Bipolar disorder	4	17.4
	Major depressive disorder	15	65.2
	Duration SMI, y, mean (SD)	26.2	12.2
Current suicide risk (MINI-plus)	No	4	17.4
	Low	6	26.1
	Medium	3	13.0
	High	10	43.5
	Suicide attempt ever	13	56.5
Current substance abuse (MINI-plus)	No	15	65.2
	Drugs	7	30.4
	Alcohol and drugs	1	4.3
Abuse, No, duration, y (LEC-5)*^c^*		Median	IQR
Childhood (< 16 year)	Emotional (*n* = 16)	8.0	5.5
	Physical (*n* = 16)	5.5	8.0
	Sexual (*n* = 12)	3.0	6.0
Adulthood (≥ 16 year)	Emotional (*n* = 11)	6.0	22.0
	Physical (*n* = 13)	3.0	8.5
	Sexual (*n* = 8)	4.5	8.0
		Mean	*SD*
Type trauma’s	Total number	8.0	1.52
PTSD (CAPS-5)^d^	Total number of symptoms	14.0	2.44
	Total severity	40.0	7.70
	Duration, y	24.1	14.48
	Dissociative subtype, No.,%	5	21.7

*^a^Mauritz et al. (19); DOI: https://doi.org/10.1192/bjo.2020.124.*

*^b^Mini-international neuropsychiatric interview.*

*^c^Life Events Checklist for DSM-5.*

*^d^Clinician-Administered PTSD Scale for DSM-5.*

**TABLE 2:** Results of primary and secondary outcomes based on a mixed model analysis for repeated measures (MMRM) of the full analysis set (FAS).^a^

Outcome		Baseline (T0)	One month after NET (T1)			Seven months after NET (T2)			Overall sig.	
		Mean (95%CI)	Mean (95%CI)	LSMD (95%CI)	*p*	Mean (95% CI),	LSMD (95%CI)	*p*	*p*	
**Primary outcomes for PTSS**										
	**CAPS-5**, total symptoms (0–20)	13.65 (11.83;15.47)	11.15 (9.25;13.04)	–2.50 (–4.19;–0.82)	0.005	8.82 (6.93;10.71)	–4.83 (–6.52;–3.14)	< 0.001	<0.001	
	**CAPS-5**, total severity (0–80)	37.91 (32.93;42.90)	31.90 (26.76;37.04)	–6.01 (–10.02;–2.01)	0.004	24.54 (19.40;29.68)	–13.37 (–17.38;–9.37)	< 0.001	<0.001	
	**CAPS-5**, total severity clusters:									
	B Intrusions (0–20)	10.78 (8.95;12.61)	9.01 (7.12;10.90)	–1.77 (–3.33;–0.22)	0.026	6.56 (4.66;8.45)	–4.23 (–5.78;–2.67)	< 0.001	<0.001	
	C Avoidance (0–8)	4.00 (3.35;4.65)	2.51 (1.82;3.21)	–1.49 (–2.38;–0.59)	0.002	1.87 (1.17;2.56)	–2.13 (–3.03;–1.24)	< 0.001	<0.001	
	D Cognitions and mood (0–28)	13.70 (11.66;15.73)	11.16 (9.04;13.29)	–2.53 (–4.52;–0.55)	0.014	9.69 (7.57;15.73)	–4.00 (–5.99;–2.02)	< 0.001	<0.001	
	E Arousal and reactivity (0–24)	9.43 (7.79;11.08)	9.01 (7.38;10.82)	–0.34 (–1.90;1.23)	0.664	6.38 (4.67;8.10)	–3.05 (–4.61;–1.49)	< 0.001	<0.001	
	**DES**, total severity (0–100)	27.79 (21.17;34.42)	22.41 (15.65;29.18)	–5.38 (–9.77;–0.99)	0.018	20.97 (14.20;27.73)	–6.83 (–11.21;–2.44)	0.003	0.008	
**Secondary outcomes for SMI**										
	**HoNOS**, total severity (0–48)	12.30 (9.79;14.81)	10.04 (7.34;12.73)	–2.27 (–4.70;0.16)	0.067	9.94 (7.31;12.54)	–2.38 (–4.72; –0.03)	0.047	0.083	
	**CAN**, total severity (0–44)	10.22 (8.21;12.22)	10.79 (8.72;12.85)	0.57 (–1.05;2.19)	0.482	9.91 (7.84;11.98)	–0.30 (–1.92;1.31)	0.705	0.562	
	**MANSA**, total quality (12–84)	52.39 (47.77;57.01)	55.38 (50.67;60.09)	2.99 (0.28;5.95)	0.048	53.04 (48.33;57.75)	0.65 (–2.31;3.61)	0.660	0.117	
	**GAF**, total functioning (0–100)	49.39 (47.00;51.78)	49.24 (46.72;51.77)	–0.15 (–2.93;2.64)	0.915	53.39 (50.86;55.91)	4.00 (1.21;6.78)	0.006	0.008	
**Care consumption*^b^***										
	**Contacts per period** (number)	35.0 (22.8;53.8)	40.0 (26.0;61.4)	5.0 (–10.8; 31.1)	0.598	30.6 (19.9;47.0)	–4.4 (–14.6;15.7)	0.597	0.572	
	**Duration of contacts** (minutes)	2021 (1,475;2,770)	3,749 (3,111;4,518)	1,728 (1,163;2,388)	< 0.001	1,271 (803;2,010)	–750 (–940; –526)	0.006	< 0.001	

*LSMD, least squares mean difference; Cl, confidence interval; CAPS-5, Clinician Administered PTSD Scale for DSM-5; DES, Dissociative Experiences Scale; HoNOS, Health of the Nation Outcome Scale; CAN, Camberwell Assessment of Needs; MANSA, Manchester Short Assessment of quality of life; GAF, global assessment of functioning scale, Number and duration of contacts from electronic record.*

*^a^Mauritz et al. (19); doi: https://doi.org/10.1192/bjo.2020.124.*

*^b^Results for the number and duration of contacts are based on analyses on a ln-scale; table present backtransformed results. The columns T0,T1 en T2 refer to the period before, during, and after treatment.*

### Qualitative Findings

#### Post-traumatic Stress Disorder Symptoms

Before NET, the participants experienced many PTSD symptoms, such as anxiety, intrusive thoughts, bad memories, nightmares, avoidance, insomnia, anger, crying, grief, guilt, negative thoughts, worrying, palpitations, pain, and fatigue.

Participants who were in remission (*n* = 11) reported that they experienced these symptoms for years and often lived a long time with it. Many of these participants experienced an increase in these symptoms during NET and reported that they were more aware of them:

During therapy: *“Experiencing grief was terrifying, allowing feelings to surface is tough and I got lost in memories. Nightmares and insomnia increased. Gradually*, *the nightmares subsided and things got better*; *I was better able to think about things*.” *Female, aged 48.*

Before and during therapy: *“I had panic attacks, nightmares, crying fits*; *I was miserable and didn’t go outside.”*

After therapy: “*I must say, things are now slowly getting better.” Female, aged 38.*

After NET, most participants reported a gradual decrease in intrusive thoughts, bad memories, nightmares, negative thoughts, insomnia, and fatigue. Although some did not notice any difference, the other 11 participants who were not in remission after NET still had PTSD symptoms, albeit, sometimes, less serious:

During therapy sessions: *“Well, the confrontation was there, but the fear was more. At the moments*, *when you talk about the abuse, I suffered from the fear for a few days[.], but then I closed myself off from it again. I had a lot of fear and reliving*.”

After therapy: *“I still have nightmares and just don’t sleep well. One minute, I have nightmares and*, *the next minute*, *I can’t fall asleep.” Female, aged 45.*

After therapy: *“I still have nightmares, but then I get out bed [sic], sit with the cat on my lap*, *and am able to sleep again. I lie awake less often now*.*” Female, aged 55.*

#### Dissociative Symptoms

Dissociative symptoms are common in PTSD but differ in severity. Thirteen participants were aware of their dissociative symptoms and told about flashbacks, reliving the memory, amnesia, absent and staring, tingling sensations, “magical thinking,” and “chaos in the head.” During and after narrative therapy, dissociative symptoms decreased in five participants and sometimes disappeared, leading to more self-knowledge and fewer symptoms, which improved the quality of life.

During and after therapy: “*Occasionally*, therapy *was a bit more intense, of course, because I noticed more about my life during the sessions, but I do notice that*, *now*, *I am just, [.] yes, much better*, *so to speak. I have no more flashbacks. I am more relaxed*; *I can put things more into perspective*, *and also just look back*.*” Female, aged 46.*

During therapy: *“You say this in the short time that you are there having a therapy session)*, *and*, *after that, you get even more awareness; often, more memories too, but also more accurate.” Male, aged 57.*

*Before therapy: “I was coming out of myself, floating around, and was magically thinking.” Afterwards*, *“I had more re-experiences.” Female, aged 45.*

#### Severe Mental Ill Symptoms

Before NET, depressive and anxiety symptoms were mentioned by most of the participants. These symptoms included feeling unwell, gloom, lots of crying, seeing everything negative, concentration problems, suicidal thoughts, insomnia, frightened feelings, and panic attacks. These symptoms led sometimes to eating problems, fatigue, and low physical health. During and after NET, 11 participants perceived less symptoms.

Before therapy: *“At the start*, *I was experiencing everything very negative.”* After therapy: “*Now, I don’t have that many crying fits anymore. I feel happy, cheerful*, *and relieved. I eat better and I look better; I am not so depressed anymore.” Female, aged 61.*

Before therapy: *“I had delusions and continued hallucinations. I was a loner, I felt tense, and I was defensive.* After therapy*: My psychotic symptoms decreased. I have more self-confidence now.” Male, aged 56.*

Five participants experienced no important changes in severe mental ill symptoms during and after NET. They told about persisting symptoms, including depressive symptoms, negative feelings, crying, insomnia, no appetite, and inability to enjoy.

After therapy: *“Nothing has changed since therapy. I have to be honest* - *I cannot control myself when it comes to eating and sleeping. And that’s because I wake up so often. Sometimes, I wake up at three o’clock. Sometimes*, *I lie down for half an hour and wake up. Then, I am sweating and trembling all over.” Male, aged 43.*

#### Care Needs

The most expressed need was good care support during NET. The participants mentioned that it was very important to have appropriate care support from trusted flexible assertive community treatment nurses, close relatives, and friends so that they could tolerate the NET. Being known, understood, and encouraged was crucial. During NET, CAN increased and were often reduced afterward, but aftercare was still needed.

*“I would say, without support, do not start, because then it has too much impact. She (nurse) came twice a week, but*, *now*, *she comes once in two weeks.” Female, aged 48.*

*“Being able to discuss things I encounter during therapy, I really needed that*.*” Female, aged 43.*

#### Quality of Life and Global Functioning

Eight participants who benefitted from NET reported an increased quality of life. Examples are feeling liberated, being more open, positive thinking, finding hobbies, allowing domestic help for oneself, handling things better, putting things in perceptive with less worrying, and trying to pick up life again.

Before therapy: *“I felt alone in the world*.”

During therapy: *“I started to think about myself differently.”*

After therapy: *“I enjoy life more*; *it doesn’t always have to go bad, and it can also go well. I have new friends*; *we do a lot together. It took a long time before I really trusted in someone.” Male, aged 56.*

After therapy: *“What’s really important is that I’ve started to allow myself more [.]. I allow myself at some point in the day to pick up a book and sit down to read or go to the village. I buy flowers and put them in a vase. Before, I wouldn’t allow myself to buy flowers while I longed for it. I have allowed myself an aquarium that had been on my wish list for decades [*…*]. Yeah, that’s just something for myself that I’d like to have.” Female, aged 52.*

Four participants who were minimally benefited or were not benefited from NET told about fewer “bad days,” living by the day, standing alone, feeling minimally good in being social, continued getting angry, missing adequate support, still having difficult days, and, sometimes, nothing was changed:

During therapy: *“I allow myself to feel bad!”* After therapy: *“My head has calmed down. I can put things into perspective and look back more relaxed.” Female, aged 46.*

After therapy: *“My life hasn’t changed. I find that very unfortunate. I would have liked that different, but it remains difficult.” Female, aged 45.*

### Mixed Methods Results

The comparison of the quantitative results based on diagnostic tools and qualitative participants’ experiences was focused on similarities and differences for post-traumatic stress disorder, dissociation, severe mental ill symptoms, CAN, quality of life, and global functioning (Table 3).

**TABLE 3:** Congruency between quantitative and qualitative data.

Quantitative assessment		One-to-one analysis of improvement during treatment period based on				Congruence	
Primary outcomes	Change of score, relative to baseline^a^	N	Quantitative instruments	Qualitative interviews		% (*p*-value)^b^	Kappa (95% CI)
			Improved	Yes	No		
PTSD	–35%***	22	Yes	11	3	73%	0.41
			No	3	5	*p* = 0.07	(0.02 to 0.81)
DES	–25%***	13	Yes	5	0	92%	0.84
			No	1	7	*p* = 0.005	(0.55 to 1.00)
**Secondary outcomes**							
HoNoS	–19%**	22	Yes	8	3	59%	0.18
			No	6	5	*p* = 0.33	(–0.21 to 0.58)
CAN	–3%	22	Yes	7	1	82%	0.63
			No	3	11	*p* = 0.005	(0.30 to 0.95)
MANSA	+1%	22	Yes	10	2	68%	0.43
			No	5	5	*p* = 0.11	(–0.04 to 0.72)
GAF	+8%***	22	Yes	12	2	68%	0.25
			No	5	3	*p* = 0.23	(–0.16 to 0.66)

^a^Difference of score between T2 versus T0, based on the quantitative strand of this mixed methods study; ***p < 0.01, **p < 0.05, *p < 0.10, two-sided ^b^P-values (one-sided) obtained from Fisher exact test.

#### Post-traumatic Stress Disorder Symptoms (CAPS-5)

Congruence was found in sixteen participants. These were participants who were in (partial) remission (*n* = 11) and those who were not in remission (*n* = 5). (Partial) incongruence was present by perceiving slight improvements (*n* = 3) and was present in two participants in remission according to the quantitative results, who expressed persistent symptoms. One participant expressed improvement, while there was hardly any change in post-traumatic stress disorder symptoms (*n* = 3).

#### Dissociative Symptoms

Thirteen participants mentioned dissociative symptoms. Congruence was found in experiencing reduced dissociative symptoms (*n* = 5) and in remaining dissociative symptoms (*n* = 7). Incongruence was present in one participant: dissociation was still present based on the DES, but the experience was different because “chaos is gone.”

#### Severe Mental Ill Symptoms

Congruence in severe mental ill symptoms was found in perceived reduced symptoms (*n* = 11) and when there are no changes (*n* = 5). (Partial) incongruence was present in perceived improvement, but not in quantitative scores symptoms (*n* = 7). In two persons, quantitative measures were improved, but they perceived no changes in symptoms.

#### Care Needs

In eighteen participants, congruence was found in perceiving no change in CAN (*n* = 11) and less CAN (*n* = 7). (Partial) incongruence consisted of improvement in CAN scores, but perceived CAN did not change (*n* = 3). In one participant, CAN scores decreased and increased over time, but perceived CAN diminished.

#### Quality of Life (MANSA)

Congruence (*n* = 15) was present in perceived better quality of life (*n* = 10) and in others who did not perceive changes (*n* = 5). Partial congruence consisted of perceived improved quality of life (*n* = 5). Two participants perceived no changes in quality of life, but MANSA scores improved.

#### Global Functioning

Congruence (*n* = 15) was present in perceived global functioning (*n* = 12) and in others who did not perceive changes (*n* = 3). Partial congruence consisted of perceived improved global functioning (*n* = 5). Two participants perceived no changes in quality of life, but GAF scores improved.

#### Congruency of the Qualitative and Quantitative Results

Table 3 summarizes the congruency between qualitative and quantitative assessments of study outcomes. Most outcomes were assessed in *n* = 22 subjects, DES in 13/22 persons. The scores decreased significantly with 25–35% for the two outcomes: PTSD and DES, whereas they changed less or did not change significantly for the other outcomes. Experienced improvement reported in the interviews was related to the changes assessed by the quantitative measurement instruments. Congruency was highest (> 73%) for PTSD, dissociative symptoms (DES), and CAN, but not for SMI (HoNoS), quality of life (MANSA), and global functioning (GAF). Cohen’s kappa was statistically significant for the qualitative and quantitative symptoms of the clinical characteristics PTSD and dissociation as well as for CAN.

## Perceived Support and Relation With Narrative Exposure Therapy Results

### Qualitative Findings

Ten out of 11 participants who were in remission for post-traumatic stress disorder perceived the professional and informal support as sufficient to very good.

*“I got enough support from the nurse. She helped me with tips for insomnia, reassured me by saying ‘this will pass’ She helped me to continue the therapy because I hesitated. I could talk about it with her and that helped. I see her as a buddy.” “My husband listened to me and my children knew what I was doing. I also received a lot of support from my boss.” Female, aged 43.*

Six participants, who were not in remission, received less support during NET because of insufficient professional or informal support.

*“I am disappointed with the mental health institute*; *I needed help but had doubts to ask for help. My supporter left the institute unexpectedly*; *he did not even say good bye! Therapy was good, but aftercare was needed and not given.” “I found support from fellow patients.” Female, aged 51.*

Five participants not in remission experienced insufficient care during and after NET. For some patients, it was not possible to contact the mental institute when needed; others had no support from the FACT during NET, and some did not get aftercare.

*“I called the crisis service, but I was not on the list. They advised to call the General Practice. I found that difficult, if you don’t get help, then you have to call a GP?” Female, aged 28.*

*“I couldn’t handle traveling by taxi, and there was no support was not organized by the therapist.” Female, aged 63.*

In addition, they had no (adequate) support from close relatives, friends, or residential caregivers.

*“My parents are not interested*; *they only speak of themselves. They never say: ‘I love you.’*

*I feel misunderstood by people.” Female, aged 28.*

About residential caregivers: *“They can’t really do anything for me other than just talk*; *it doesn’t solve the problem.” Female, aged 27.*

### Pairwise Analysis of Support and Remission

Clinical remission of PTSD, assessed by the diagnostic instrument, was strongly related to perceived support during the treatment period (Table 4). All the patients who perceived sufficient to very good professional and informal support (10/10) were in remission at T2 (quantitative assessment). Among the 12 patients who experienced less or no support, only one (9%) was in remission (*p* < 0.0001, Fisher exact test).

**TABLE 4:** Association between experienced support during NET and remission of PTSD.

Remission PTSD at T2 (PTSD questionnaire)	Experienced support (interviews)		N
	Sufficient to very good	Insufficient or not	
Yes	10 (100%)	1 (9%)	11
No	0	11	11
Total	10	12	22

*Difference between 100 and 9% statistically significant p < 0.0001 (one-sided, Fisher exact test).*

## Discussion

To our knowledge, this is the first mixed methods study investigating the efficacy and the experiences of NET in outpatients with SMI with comorbid PTSD receiving community mental healthcare in flexible assertive community treatment teams.

The results of the qualitative and mixed methods analysis indicated that the participants lived with PTSD for many years. The most frequently mentioned symptoms included avoidance, intrusive thoughts, nightmares, anxiety, depression, suicidality, and low physical health. Receiving NET was an intensive experience but was mostly bearable. During NET, most of the participants experienced fewer symptoms. Perceived post-traumatic stress disorder and dissociation decreased, leading to slight improvement in quality of life. After NET, two-thirds of the participants perceived less of these symptoms. The main expressed care need was appropriate support to tolerate NET. Being known, understood, and encouraged was crucial. The participants who benefited from NET experienced increased quality of life and functioning. Those who did not benefit, often lacking proper care support, were less good at being social, and experienced more negative emotions.

The mixed methods results showed that the qualitative experiences were congruent with diagnostic scores for PTSD, dissociative symptoms (DES), and CAN (*p* < 0.05, one-sided). Some participants perceived improvement with respect to SMI symptoms, but this was not congruent with the scores of the HoNOS (Table 3). In the quantitative analysis, however, SMI symptoms as assessed by HoNOS were significantly reduced after NET (Table 2). The participants who indicated that they experienced sufficient support were more often in remission than those who did not experience support. Qualitative experiences for quality of life and global functioning were not congruent with quantitative scores (MANSA and GAF).

### Results in Context

The participants in this study experienced NET as intense, but most of them were able to tolerate it and completed the therapy. Until recently, NET was not offered to outpatients with SMI with comorbid post-traumatic stress disorder, because many mental health practitioners and researchers were convinced that TFT like NET for these vulnerable patients would not be tolerated. Moreover, as is the case in most studies concerning TFT, NET studies often excluded patients with psychotic disorder, bipolar disorder, substance misuse, and severe suicidal ideations (15, 39–41).

This exclusion is often based on the clinical perception of vulnerability in patients with SMI, which is long-standing and resulted often in inappropriate psychiatric care, such as avoidance of trauma and overly caring behavior and leads to undertreatment (19). A recent meta-analysis of TFT for post-traumatic disorder in patients with SMI has shown that PTSD treatments had a large effect on PTSD outcomes. Still, it was also concluded that patients with SMI often do not receive evidence-based TFT (42). Unfortunately, resilience is still underestimated in outpatients with SMI (43) and also in patients with post-traumatic stress disorder (44).

### Symptom

This study showed that NET is clearly efficacious in outpatients with SMI with comorbid post-traumatic stress disorder and dissociation. Qualitative findings confirm these results and also clarified which trauma symptoms bothered participants the most.

In the Netherlands, Routine Outcome Monitoring for patients with SMI is periodically performed with the HoNOS for severe mental ill symptoms, CAN for CAN, and MANSA for quality of life. These instruments were used for SMI, but outcomes were hardly significant for SMI or CAN and quality of life in this group (19). Qualitative results confirm that severe mental ill symptoms decreased slightly and that CAN hardly changed, but the participants mentioned other specific CAN, and quality of life slightly improved.

One can question if these instruments are responsive enough to evaluate the treatment effect on severe mental indicators. Because this population has different symptom burdens, generic measuring instruments are not distinctive enough to see the specific effects of treatment. Disorder-specific measures have the most precise responsiveness for individual treatment and are recommended for clinical use (45). Therefore, a structured diagnostic interview, such as the M.I.N.I.-plus (46), provides more precise information for different psychiatric diagnoses and is also suitable because of generic items, such as substance abuse.

### The Importance of Adequate Professional and Informal Support

Outpatients with SMI with comorbid post-traumatic stress disorder are vulnerable, but they can tolerate exposure if there is sufficient informal and professional support. It was striking that those who actually experienced informal social support and adequate professional support were able to benefit more from NET than those who missed adequate informal or professional support or both. In some cases, it turned out that the protocol agreement to offer support at least every 2 weeks was not kept. It is, therefore, clear that careful adherence to the treatment protocol must be maintained.

Social relationships play a crucial role in recovery from trauma (47), and high informal social support may protect against the negative effects of PTSD (48). Mueser et al. (49) also argued that traumatized patients with SMI who received social support have a better course of illness, which can minimize the effects of stress (49). Furthermore, positive support can provide a foundation for resilience because it reduces stress levels, depression, and (severity of) post-traumatic stress disorder (44). Our study confirms these results because there was a clear link with adequate informal and professional support and the effect of NET. Flexible assertive community treatment team members in this study provided psychiatric supportive care and also, often, social support, especially in the patients who hardly had a social network.

### Strengths

The first strength of this study was that it was conducted in the real-life clinical context. As a result, there is less chance of overestimating the effect of the intervention. Second, NET was provided by trained and certified therapists, who were all flexible assertive community treatment team members and, therefore, familiar with patients with SMI. Third, NET fitted well in the workflow of the involved professional, which is helpful for future implementation. Fourth, quantitative results were firm, despite being a small group (19). Fifth, the qualitative analysis based on QUAGOL stages was done very thoroughly and included *peer debriefing, transparency and reproducibility, and triangulation*. Based on feedback from all the authors, c*onstant comparison* and *thematic analysis* were undertaken. Sixth, after 22 interviews with enriching data, saturation was reached. Seventh, the mixed analysis showed the important correspondences and differences between quantitative diagnostic results and participants’ perceptions. Congruence and incongruence were both present, reflecting daily clinical practice. Eighth, the relationship between supportive care and being able to benefit from NET was convincing. Lastly, this mixed methods design gave more in-depth knowledge and insights.

### Limitations

The first limitation of this study is that the results were based on a small group of patients, which restricts generalization and holds especially true for the few men who participated in the study. Second, there was no control group; therefore, the results may not point at a causal effect of NET. Third, flexible assertive community treatment team members selected patients with possible post-traumatic stress disorder from their own case load who could potentially benefit from NET. This may have led to selection bias and reduced representativeness for the entire population in flexible assertive community treatment teams. Therefore, screening for trauma exposure and post-traumatic stress disorder was carefully performed with appropriate instruments: LEC-5 (50) and PCL-5 (51).

### Implications

This study shows that patients with SMI with comorbid post-traumatic stress disorder can be treated well with NET and that this therapy was highly applicable in this sample of patients with SMI. Although it was a small study population, results were convincing. Given the similarities with other TFT, it is plausible that NET can also be offered to patients with SMI (11, 42).

Patients with SMI and comorbid post-traumatic stress disorder often have a history of abuse and neglect, both in the family of origin and by mental healthcare providers based on incorrect assumptions (2, 11, 52, 53). Actual severe mental ill symptoms hinder to assertively ask for adequate help. Moreover, most of the patients with SMI also have comorbid somatic disorders related to the side effects of psychotropic drugs and an unhealthy lifestyle, which possibly reinforces further social marginalization (54, 55). These factors can also lead to pharmaceutical undertreatment or overtreatment (56). To counteract this, regular screening of present post-traumatic stress disorder, other comorbid mental disorders, drug use, and somatic disorders is necessary. Simultaneous adequate care support is an important condition for the effectiveness of the entire treatment and promotes resilience. During intensive therapies such as NET, more support is needed than usual in this specific group.

## Conclusion and Recommendations

In our study, NET is clearly efficacious in outpatients with SMI and comorbid post-traumatic stress disorder. TFT such as NET should certainly be actively offered to this specific vulnerable population, because the prevalence is high, and chronic trauma-related symptoms negatively affect the main disorder and overall functioning. In addition, it is important that the resilience of patients with SMI is not underestimated, but optimal professional and informal support are necessary to strengthen this resilience. Therefore, prior to intensive trauma treatment and in consultation with the patient, it is recommended to involve professional and informal caregivers. Training and equipping flexible assertive community treatment team members are also recommended, especially for nurses, who often have intensive contact with patients with SMI and usually are the first to observe changes during NET on which they can give quick and adequate care support.

Because this was a small group with no control, in the future, a controlled study on NET with patients with SMI is recommended.

## Data Availability Statement

The original contributions presented in the study are included in the article/Supplementary Material, further inquiries can be directed to the corresponding author/s.

## Ethics Statement

The studies involving human participants were reviewed and approved by the Committee on Research Involving Human Subjects Arnhem-Nijmegen provided ethical approval (no. 1843–2015). The patients/participants provided their written informed consent to participate in this study.

## Author Contributions

MM conducted the interviews and the qualitative and mixed methods analyses, drafted, and revised the manuscript. PG and MM conducted the qualitative analysis together and contributed to the mixed methods analysis. RJ provided expertise on narrative exposure therapy and psychiatric treatment for vulnerable traumatized people. HV supervised the study and contributed to critical evaluation of the qualitative and mixed research. BG contributed with her expertise in qualitative and mixed methods analyses and provided constructive feedback. All authors contributed to drafting and revision of the manuscript.

## Conflict of Interest

The authors declare that the research was conducted in the absence of any commercial or financial relationships that could be construed as a potential conflict of interest.

## Publisher’s Note

All claims expressed in this article are solely those of the authors and do not necessarily represent those of their affiliated organizations, or those of the publisher, the editors and the reviewers. Any product that may be evaluated in this article, or claim that may be made by its manufacturer, is not guaranteed or endorsed by the publisher.
